# Sex differences in major adverse cardiovascular and cerebrovascular event risk among central retinal artery occlusion patients

**DOI:** 10.1038/s41598-023-42247-2

**Published:** 2023-09-11

**Authors:** Ting Chen, Yuedan Wang, Xuejie Li, Jiaqing Feng, Hongxia Yang, Ying Li, Hui Feng, Xuan Xiao

**Affiliations:** 1 Department of Ophthalmology, Renmin Hospital of Wuhan University, No. 238 Jie Fang Road, Wuhan, 430060 Hubei China; 2 Information Center, Renmin Hospital of Wuhan University, No. 238 Jie Fang Road, Wuhan, 430060 Hubei China

**Keywords:** Retinal diseases, Cardiovascular diseases, Randomized controlled trials

## Abstract

To estimate the association between central retinal artery occlusion (CRAO) and major adverse cardiovascular and cerebrovascular events (MACCE), including their clinical characteristics, blood markers, and the contribution of CRAO to MACCE, as well as to assess any sex differences. This retrospective cohort study included continuous new-onset CRAO patients and 1:4 controls during the same period. Correlations of CRAO with the incidence of MACCE during follow-up and the sex-related differences were studied. One hundred and twenty-four CRAO patients and four hundred and ninety-six controls were enrolled. Neutrophil-to-lymphocyte ratio (NLR, P = 0.014) and high-sensitivity C-reactive protein (hs-CRP, P = 0.038) were tended to be higher in CRAO patients. After the follow-up period, 78 patients experienced MACCE. Multivariate Cox regression analysis showed that CRAO was a predictor of the occurrence of MACCE (HR 2.321, 95% CI 1.439–3.744, P = 0.001). Sex subgroups indicated that age, diabetes, current smoking, CRAO, NLR and hs-CRP increased the risk factor of MACCE in males (All P < 0.05) and CRAO, NLR, low-density lipoprotein cholesterol (LDL-C) and hs-CRP were independent influencing factors for females (All P < 0.05). New-onset CRAO significantly increases the probability of MACCE and is associated with a poor prognosis. The sex-related differences suggested that effective prevention of the occurrence of MACCE in high-risk patients requires that attention be given to individualized risk factors corresponding to sexes.

## Introduction

As an emergency ophthalmic disease, retinal artery occlusion (RAO) frequently results in sudden and severe loss of vision^1^. RAO can be roughly divided into central retinal artery occlusion (CRAO) or branch retinal artery occlusion (BRAO), depending on the location of the lesion^2^. Currently, there is accumulating evidence to support CRAO as a potential risk factor for cardiovascular or cerebrovascular diseases such as stroke, coronary artery disease, and atrial fibrillation^3–5^. Notably, patients with sudden-onset CRAO have a higher risk of developing ischemic stroke within a few weeks of onset^4–7^. Moreover, patients with RAO were more likely to experience acute myocardial infarction (AMI)^5^ and atrial fibrillation and arrhythmia^8^, suggesting that RAO, especially CRAO, might be a risk factor of underlying cardiovascular disease. Pathologically, RAO and cardiovascular and cerebrovascular diseases are characterized by progressive atherosclerosis, which may suggest a common underlying pathophysiological basis. Therefore, it is important for clinicians to thoroughly follow-up with cardiovascular examinations and medical prevention for patients following RAO.

Strikingly, there are significant differences between male and female in the presentation, risk factors and treatment of cardiovascular and cerebrovascular disease^9^. For example, previous studies demonstrated that females are less likely to present with acute coronary syndromes than males^10,11^; however, females are much more likely to develop coronary microvascular dysfunction without angiographically obstructive coronary artery disease (CAD)^12^. Moreover, males and females differ with regard to key features of heart failure^13–15^.

Although the relationship between RAO and cardiovascular and cerebrovascular disease has been studied, previous studies have rarely quantified classical cardiovascular risk factors and medications, and fewer studies have analyzed important parameters of blood cell counts and biochemical parameters. Furthermore, to the best of our knowledge, there remains no literature providing any evidence that the incidence of major adverse cardiovascular and cerebrovascular events (MACCE) associated with CRAO differ between sexes. The objective of this study was to investigate whether CRAO could serve as a predictor of the incidence and poor prognosis of MACCE in the overall population and to perform sex subgroup analyses. In this study, we found that New-onset CRAO significantly increases the probability of MACCE and is associated with a poor prognosis. The sex-related differences suggested that effective prevention of the occurrence of MACCE in high-risk patients requires that attention be given to individualized risk factors corresponding to sexes.

## Materials and methods

### Study population

**Figure 1:**
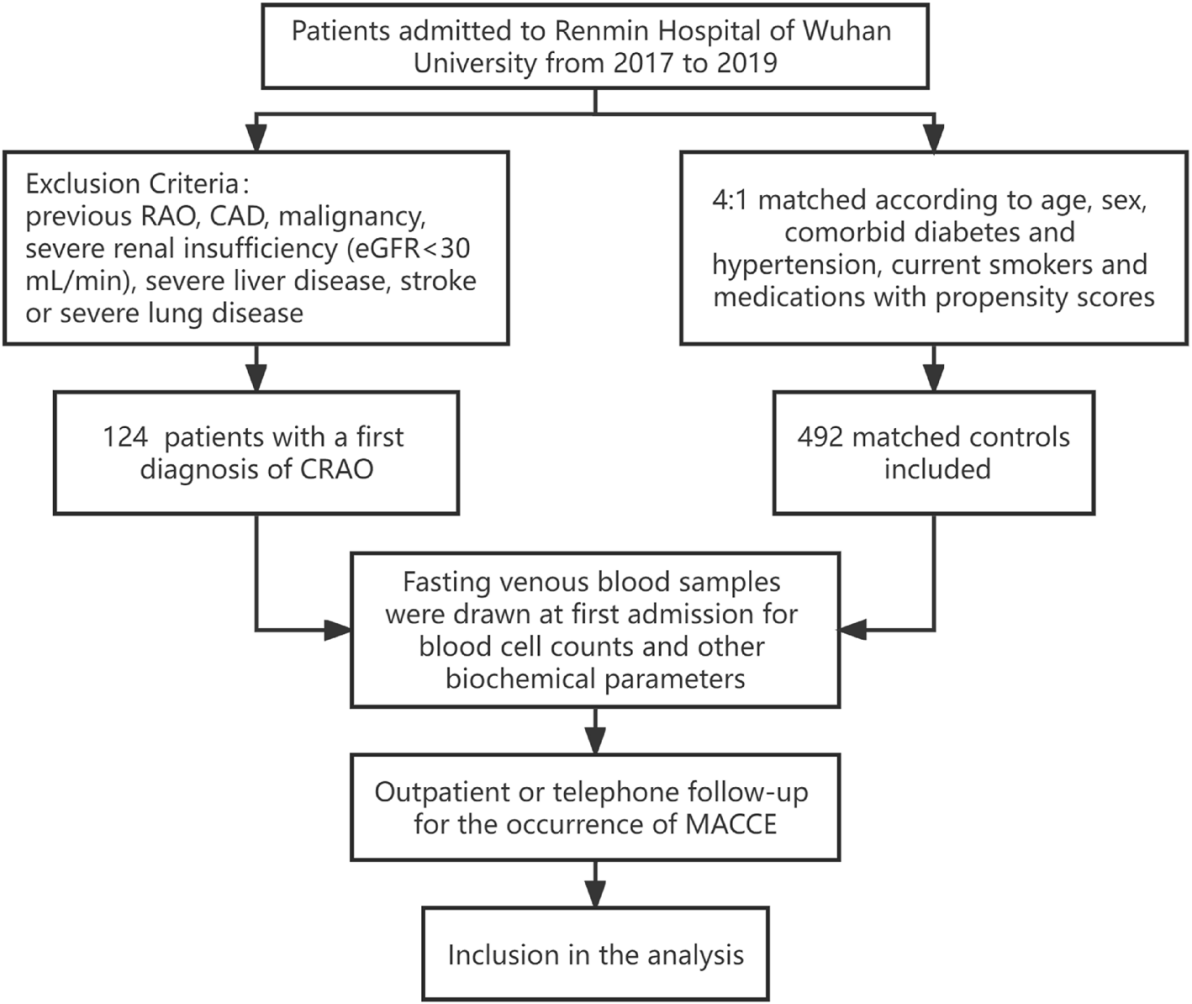
The workflow of participant inclusion and exclusion criteria.

A single-center retrospective cohort study was launched at the Renmin Hospital of Wuhan University from 2017 to 2019. The workflow of participant inclusion and exclusion criteria were shown in Fig. 1. A total of 124 consecutive patients with a first diagnosis of CRAO. The diagnosis of CRAO was in accordance with the previously published guidelines^16^, including a sudden loss of visual acuity (VA), positive relative afferent pupillary defect (RAPD), retinal ischemic edema and delayed arteria filling on fluorescein angiography (FA). CRAO patients with any known history of the following conditions were ruled out: previous RAO, CAD, malignancy, severe renal insufficiency (eGFR < 30 mL/min), severe liver disease, stroke or severe lung disease. Controls are defined as individuals without RAO history who underwent coronary angiogram to rule out CAD during the same period (patients with a subsequent diagnosis of CAD were included). Control group (n = 496) were matched to included CRAO patients at a 4:1 ratio based on age, sex, comorbid diabetes and hypertension, current smokers and medications with propensity scores matching method. The baseline demographics, clinical characteristics and therapeutic applications of all individuals were retrieved from patient medical records. Hypertension patients were defined as who were taking any antihypertensive medication to control their blood pressure or with blood pressure over 140/90 mmHg measured on two or more separate occasions^17^. Patients with diabetes history or whose measured fasting and/or postprandial blood glucose exceeded the standards set by guidelines were defined as diabetes patients^18^. Smoking/drinking status was divided into no (including never and past smokers/drinkers) and current smokers/drinkers. Stroke was defined as fatal or non-fatal ischemic stroke. Our study was approved by the Ethical Committee Board of Renmin Hospital of Wuhan University (approval number WDRY2022-K278) and followed the tenets of the Declaration of Helsinki. As this was a retrospective observational study, the study protocol was approved, and the requirement for the informed consent from eligible patients was waived.

### Blood sampling and definitions of MACCE risk factors

As a routine part of our clinical practice, venous blood sample was drawn from patients after overnight fasting in the morning at first admission and analyzed for blood cell counts and other biochemical parameters. Parameters of blood cell count included leukocyte parameters (counts of white blood cell [WBC], neutrophils and lymphocytes and neutrophil-to‐lymphocyte ratio [NLR]) and platelet parameters (count, mean volume [MPV] and distribution width [PDW] of platelets, and platelet-to-lymphocyte ratio [PLR]). Biochemical parameters consisted of renal function parameters (creatinine, estimated glomerular filtration rate [eGFR] and uric acid [UA]), glucose, lipid profile (triglycerides [TGs], total cholesterol [TC], high-density [HDL-c] and low-density lipoprotein cholesterol [LDL-C], apolipoprotein A1 [ApoA1] and B [Apo B], and lipoprotein a), inflammatory markers (hypersensitive C-reactive protein [hs-CRP]), and liver function parameters (fibrinogen, total bilirubin [TBil] and direct bilirubin [DBil]).

### Follow-up

After discharge, follow-up of patients was performed via outpatient visits or telephone for 3 years. Oral informed consent was obtained after explanation of the nature and possible consequences of our study. The clinical endpoint of this research was defined as the occurrence of MACCE, consisting of all-cause mortality, cardiac mortality, acute coronary syndromes (ACS), stroke, revascularization and admission to the hospital necessitated by heart failure (HF) or atrial fibrillation (AF). Two experienced physicians adjudicated the endpoint events based on medical record reviewing. All-cause mortality was defined as the death from all causes. Cardiac mortality was defined as overall mortality from cardiac causes. ACS was defined as a novel ACS in the target vessel and confirmed by an angiography. Revascularization was defined as revascularization on target vessels. Hospitalization for HF or AF was defined as admission for worsening signs or symptoms of HF or AF resulting in the augmentation of HF or AF therapies.

### Statistical methods

Mean and standard deviation (SD) or median and interquartile range (IQR) were adopted to express continuous variables, and percentages are used to present categorical variables. The chi-square (χ^2^) test was employed to analyze the categorical variables. Kaplan–Meier survival analysis was employed to understand the prognostic factors for MACCE. First, univariate Cox analysis was executed to identify significant variables, and then these variables were included in the multivariate Cox analysis. To avoid collinearity, we used NLR instead of neutrophil count or lymphocyte count in multivariate analysis. Hazard ratio (HR) and 95% confidence interval (CI) were calculated. A P value < 0.05 indicated a statistically significant difference. SPSS 23.0 (SPSS Inc, Chicago, IL, USA) was used for all analyses.

### Institutional review board statement

The study was conducted according to the guidelines of the Declaration of Helsinki, and approved by the Ethical Committee Board of Renmin Hospital of Wuhan University (protocol code, WDRY2022-K278 and approval date, 30 November 2022).

### Informed consent statement

Since data were evaluated retrospectively, pseudonymously and were solely obtained for treatment purposes, a requirement of informed consent was waived by the Ethical Committee Board of Renmin Hospital of Wuhan University (approval number WDRY2022-K278).

## Results

### Characteristics of the control group and CRAO group

Table 1 showed that neutrophil count (P = 0.038), NLR (P = 0.014), creatinine (P = 0.049), UA (P = 0.002), TG (P = 0.001), TC (P < 0.001), LDL-C (P < 0.001), Apo B (P < 0.001), hs-CRP(P = 0.038), TBil (P < 0.001) and DBil (P = 0.029) were significant higher in CRAO patients than in control group, while mean platelet volume were significant lower in CRAO patients (P < 0.001).

**Table 1:** Characteristics of central retinal artery occlusion group and control group.

	Control group (n = 496)	CRAO group (n = 124)	t/Z/χ^2^	P
Sex (male%)	262 (52.8)	67 (54.0)	0.058	0.809
Age (years)	61.57 ± 9.33	61.62 ± 11.66	0.051	0.959
Hypertension (%)	223 (45.0)	55 (44.4)	0.015	0.904
Duration of Hypertension (years)	8.00 (4.00, 11.00)	7.00 (4.00, 10.00)	0.237	0.813
Diabetes (%)	58 (11.7)	21 (16.9)	2.452	0.117
Duration of Diabetes (years)	5.50 (2.00, 10.00)	8.00 (2.50, 10.50)	0.472	0.637
Current smoker (%)	110 (22.2)	24 (19.5)	0.413	0.521
Duration of smoking (years)	30.00 (20.00, 30.00)	30.00 (12.25, 30.00)	0.570	0.569
Current smoking cigarettes (per day)	20.00 (10.00, 20.00)	15.50 (8.00, 20.00)	1.638	0.101
Current drinker (%)	59 (11.9)	10 (8.1)	1.472	0.225
Fatty liver (%)	11 (2.2)	5 (4.0)	0.678	0.410
WBC (× 10^9^/L)	5.99 ± 1.56	6.09 ± 1.85	0.615	0.539
Neutrophil (× 10^9^/L)	3.64 ± 1.29	3.92 ± 1.49	2.080	0.038*
Lymphocyte (× 10^9^/L)	1.78 ± 0.57	1.69 ± 0.61	1.583	0.114
NLR	1.94 (1.50, 2.60)	2.18 (1.63, 2.94)	2.461	0.014*
PLT (× 10^9^/L)	214.95 ± 58.50	208.80 ± 56.86	1.053	0.293
MPV (fL)	11.35 ± 2.05	10.87 ± 1.05	3.605	< 0.001*
PDW (%)	12.77 ± 2.36	12.79 ± 2.54	0.091	0.928
PLR	129.35 ± 45.64	136.64 ± 55.51	1.352	0.178
Creatinine (μmol/L)	66.93 ± 16.98	71.32 ± 23.17	1.981	0.049*
eGFR (mL/min)	92.89 ± 14.43	92.97 ± 18.42	0.050	0.960
UA (μmol/L)	364.22 ± 91.99	394.11 ± 104.22	3.149	0.002*
Glucose (mmol/L)	5.43 ± 1.50	5.23 ± 1.27	1.370	0.171
TG (mmol/L)	1.38 (1.01, 1.98)	1.61 (1.25, 2.35)	3.378	0.001*
TC (mmol/L)	4.40 ± 1.04	4.76 ± 1.05	3.514	< 0.001*
HDL-C (mmol/L)	1.17 ± 0.30	1.13 ± 0.31	1.131	0.258
LDL-C (mmol/L)	2.38 ± 0.80	2.73 ± 0.87	4.255	< 0.001*
Apo A1 (g/L)	1.35 ± 0.20	1.35 ± 0.23	0.062	0.951
Apo B (g/L)	0.88 ± 0.19	0.95 ± 0.20	3.591	< 0.001*
Lp (a) (g/L)	146.00 (71.00, 343.88)	143.50 (80.00, 362.45)	0.196	0.845
hs-CRP (mg/L)	0.83 (0.26, 2.35)	1.20 (0.32, 3.73)	2.072	0.038*
Fibrinogen (g/L)	2.90 ± 0.77	2.97 ± 0.79	0.869	0.385
TBil (μmol/L)	12.73 ± 5.71	15.15 ± 6.32	4.132	< 0.001*
DBil (μmol/L)	3.85 ± 1.91	4.28 ± 2.15	2.184	0.029*
Medications (%)				
Aspirin	317 (63.9)	73 (58.9)	1.080	0.299
Statins	371 (74.8)	89 (71.8)	0.474	0.491
β-Blocker	115 (23.2)	28 (22.6)	0.020	0.886
ACEI/ARB	52 (10.5)	15 (12.1)	0.268	0.605
CCB	106 (21.4)	26 (21.0)	0.010	0.922
MACCE (%)	49 (9.9)	29 (23.4)	16.458	< 0.001*
All-cause death	1 (0.2)	2 (1.6)	1.696	0.193
Cardiac death	1 (0.2)	1 (0.8)	0.031	0.859
Revascularization	3 (0.6)	4 (3.2)	3.982	0.046*
Stroke	12 (2.4)	12 (9.7)	12.161	< 0.001*
Acute coronary syndromes	35 (7.1)	18 (14.5)	7.061	0.008*
Atrial fibrillation	11 (2.2)	4 (3.2)	0.107	0.744
Heart failure	5 (1.0)	0 (0.0)	0.315	0.575

*WBC* white blood cell count, *NLR* neutrophil‑to‑lymphocyte ratio, *PLT* platelet count, *MPV* mean platelet volume, *PDW* platelet distribution width, *PLR* platelet‑to‑lymphocyte ratio, *eGFR* estimated glomerular filtration rate, *UA* uric acid, *TG* triglycerides, *TC* total cholesterol, *HDL-C* high-density lipoprotein cholesterol, *LDL-C* low-density lipoprotein cholesterol, *Apo A1* Apolipoprotein A1, *Apo B* Apolipoprotein B, *Lp (a)* lipoprotein a, *hs-CRP* hypersensitive C-reactive protein, *TBil* total bilirubin, *DBil* direct bilirubin, *ACEI* angiotensin converting enzyme inhibitor, *ARB* angiotensin receptor blockers, *CCB* calcium ion channel blockers, *CRAO* central retinal artery occlusion, *MACCE* major adverse cardiovascular and cerebrovascular events. *Indicates P < 0.05.

### Predictors of clinical endpoints

The incidence of clinical outcomes for all patients during the mean follow-up period of 33.23 months in this study is shown in Table 1. During the follow-up period, 78 patients experienced MACCE. CRAO patients had a higher incidence of MACCE (P < 0.001), revascularization (P < 0.046), stroke (P < 0.001), and ACS (P = 0.008), while the differences in other parameters between the CRAO and control groups were not significant (all P > 0.05). According to Kaplan–Meier analysis, the incidence of MACCE in CRAO patients was significantly different than that in the control group (χ^2^ = 19.552, P < 0.001).

**Figure 2:**
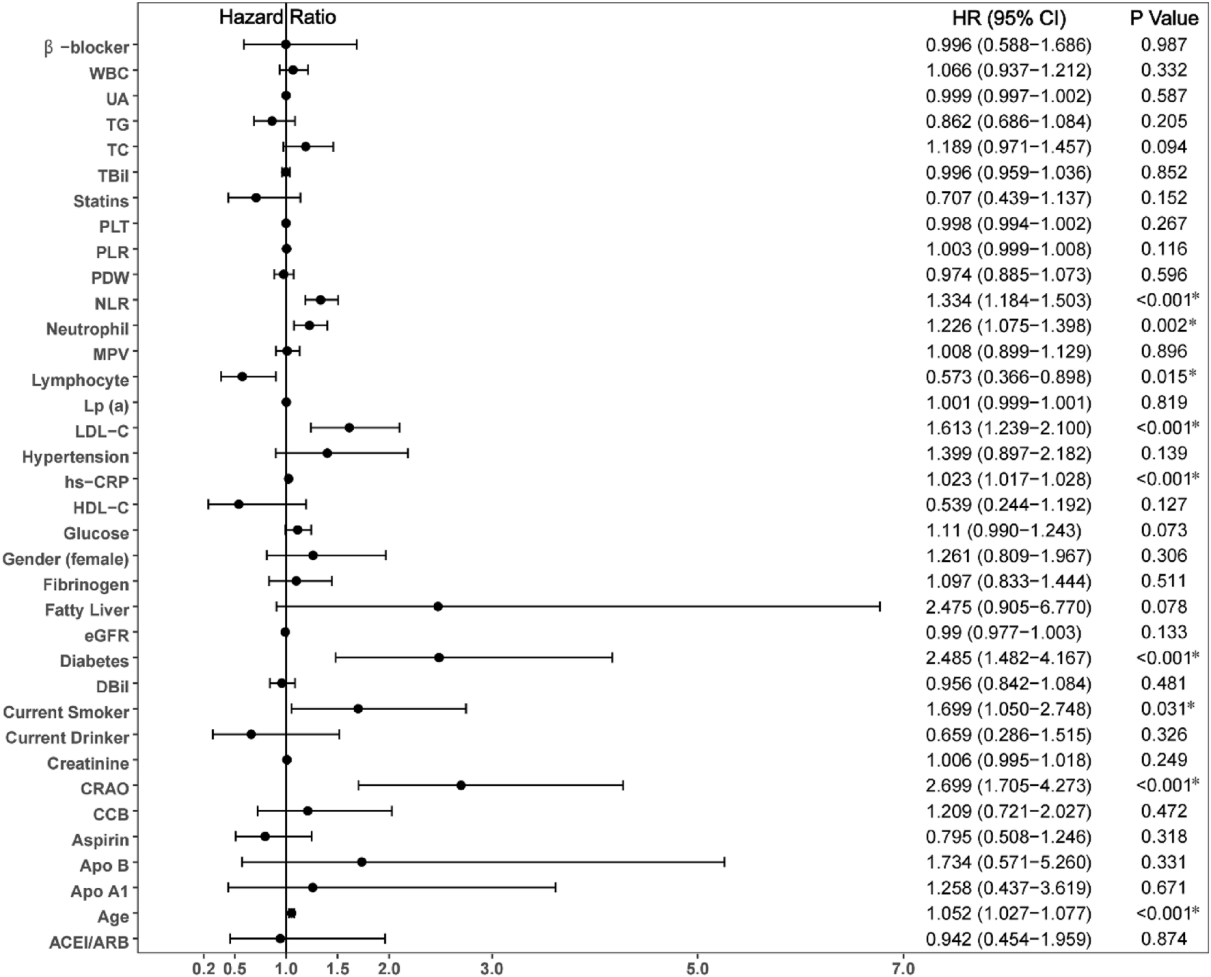
Predictors of the occurrence of major adverse cardiovascular and cerebrovascular events: results of univariate Cox-regression analyses. *Indicates P < 0.05.

**Figure 3:**
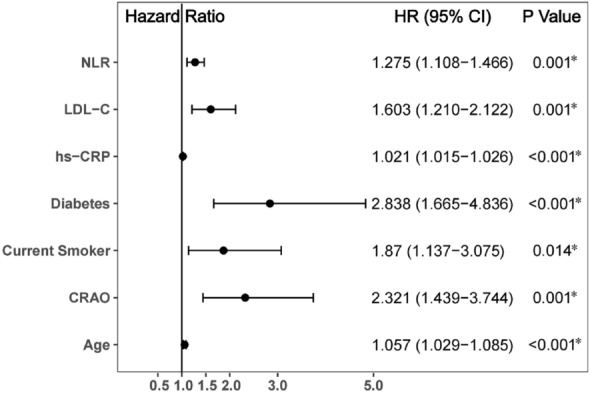
Predictors of the occurrence of major adverse cardiovascular and cerebrovascular events: results of multivariable Cox-regression analyses. *Indicates P < 0.05.

Univariate Cox analysis showed that age, diabetes, current smoking status, CRAO, neutrophil count, NLR, LDL-C and hs-CRP (all P < 0.05) were predictors of MACCE (Fig. 2). In multivariate Cox analysis, age (HR 1.057, 95% CI 1.029–1.085, P < 0.001), diabetes (HR 2.838, 95% CI 1.665–4.836, P < 0.001), current smoking (HR 1.870, 95% CI 1.137–3.075, P = 0.014), CRAO (HR: 2.321, 95% CI: 1.439–3.744, P = 0.001), NLR (HR 1.275, 95% CI 1.108–1.466, P = 0.001), LDL-C (HR 1.603, 95% CI 1.210–2.122, P = 0.001) and hs-CRP (HR 1.021, 95% CI 1.015–1.026, P < 0.001) were independent risk factors for MACCE. Additionally, for all of the evaluated patients, a potential interaction between sex and CRAO was evaluated by incorporating an interaction term into the final model obtained through multivariable Cox regression analysis (Fig. 3). The sex*CRAO interaction term was an independent influencing factors for MACCE (HR 1.547, 95% CI 1.150–2.082, P = 0.004).

### Predictors of clinical endpoints in male patients

According to Kaplan–Meier analysis, in all of the evaluated male patients, CRAO patients tended to have a higher risk of developing future MACCE than controls (χ^2^ = 8.920, P = 0.003).

**Figure 4:**
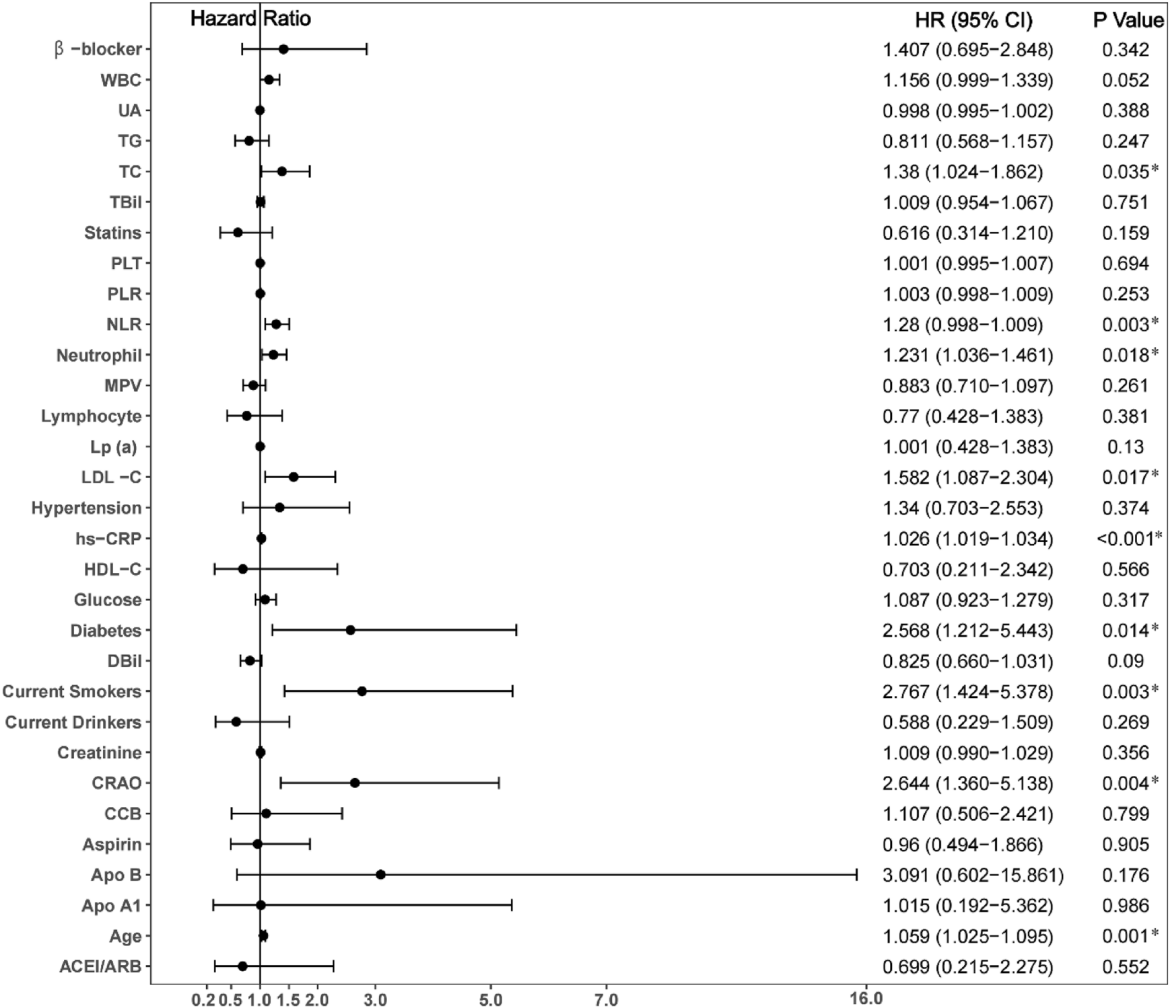
Predictors of the occurrence of major adverse cardiovascular and cerebrovascular events in male patients: results of univariate Cox-regression analyses. *Indicates P < 0.05.

**Figure 5:**
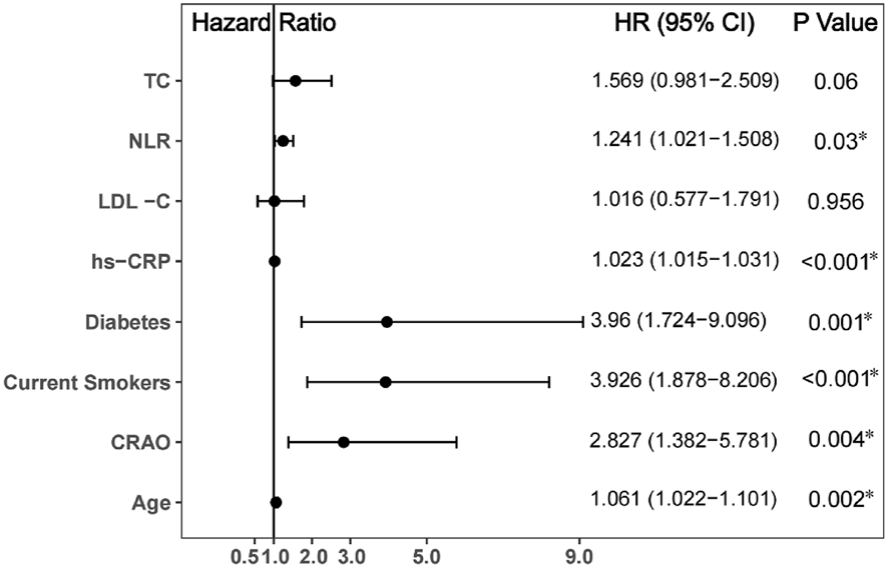
Predictors of the occurrence of major adverse cardiovascular and cerebrovascular events in male patients: results of multivariable Cox-regression analyses. *Indicates P < 0.05.

Univariable Cox analysis demonstrated that age, diabetes, current smoking status, CRAO, neutrophil count, NLR, TC, LDL-C and hs-CRP (all P < 0.05) were predictors of MACCE (Fig. 4). In addition, in all evaluated male patients, independent influencing factors for MACCE including age (HR 1.061, 95% CI 1.022–1.101, P = 0.002), diabetes (HR 3.960, 95% CI 1.724–9.096, P = 0.001), current smoking (HR 3.926, 95% CI 1.878–8.206, P < 0.001), CRAO (HR 2.827, 95% CI 1.382–5.781, P = 0.004), NLR (HR 1.241, 95% CI 1.021–1.508, P = 0.030) and hs-CRP (HR 1.023, 95% CI 1.015–1.031, P < 0.001) according to multivariate Cox analysis (Fig. 5).

### Predictors of clinical endpoints in female patients

According to Kaplan–Meier analysis, in all evaluated female patients, CRAO patients tended to have lower MACCE-free survival rates than controls (χ^2^ = 10.899, P = 0.001).

**Figure 6:**
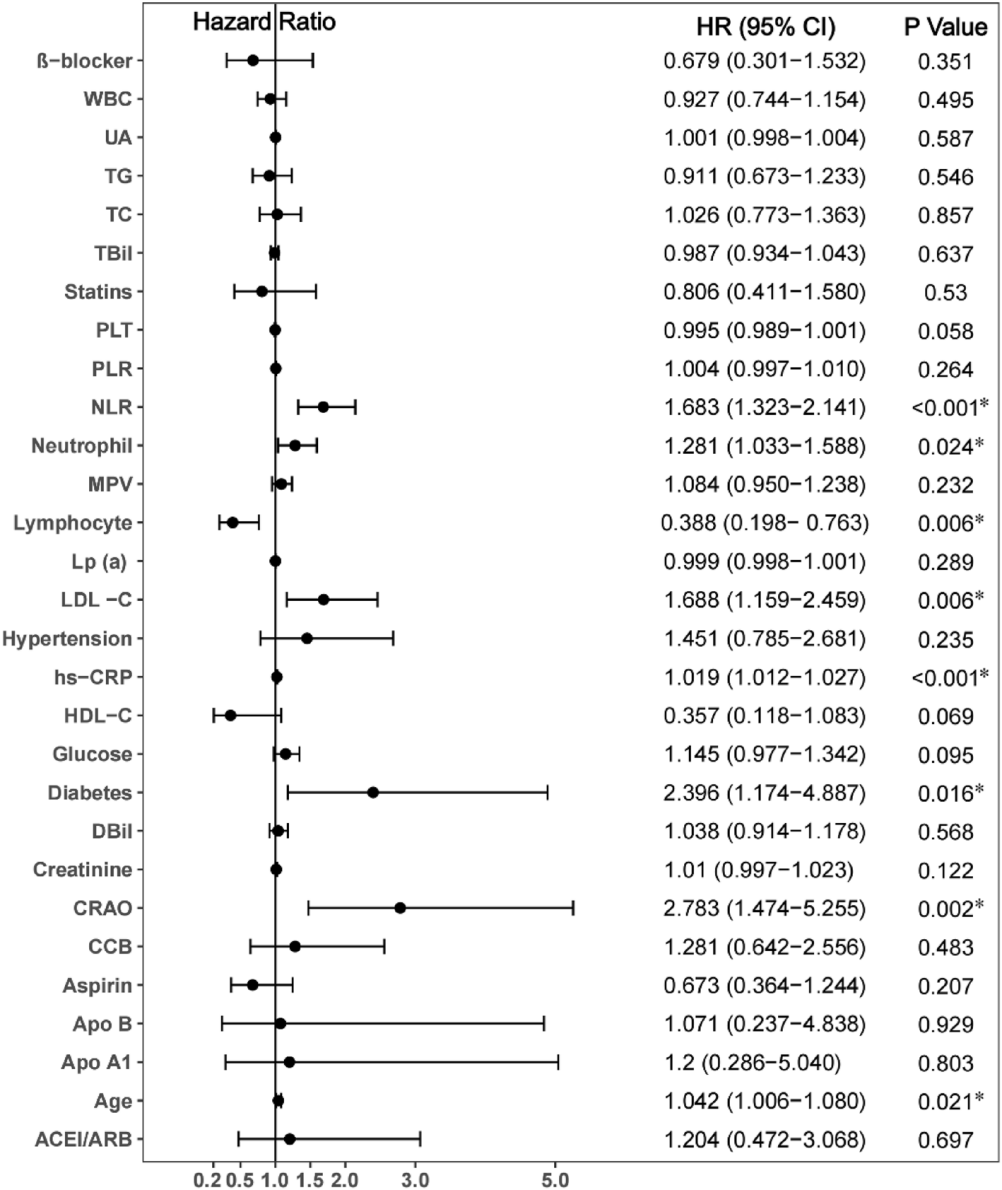
Predictors of the occurrence of major adverse cardiovascular and cerebrovascular events in female patients: results of univariate Cox-regression analyses. *Indicates P < 0.05.

**Figure 7:**
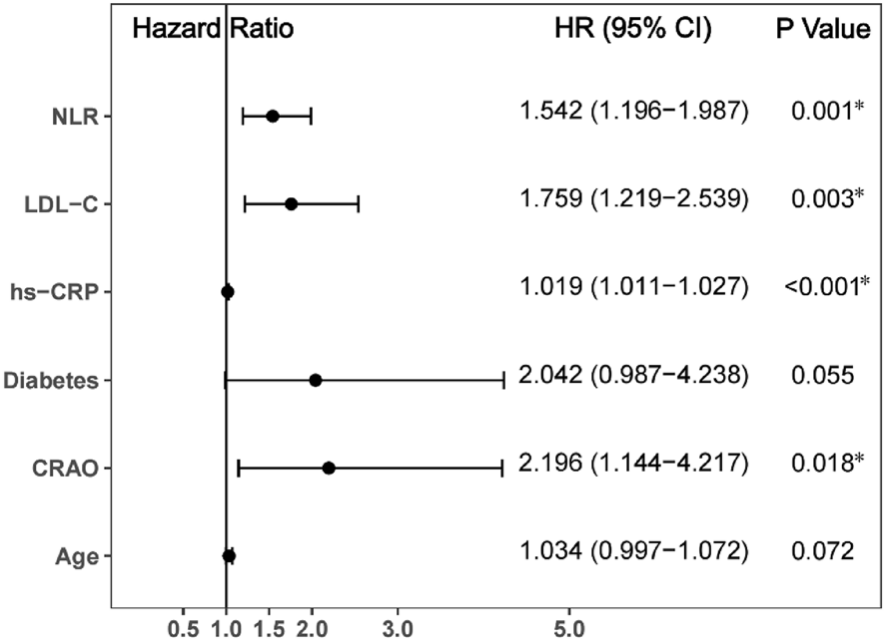
Predictors of the occurrence of major adverse cardiovascular and cerebrovascular events in female patients: results of multivariable Cox-regression analyses. *Indicates P < 0.05.

Age, diabetes, CRAO, neutrophil count, lymphocyte count, NLR, LDL-C, and hs-CRP were predictors of MACCE in univariate Cox analysis (Fig. 6) (all P < 0.05). In all of the evaluated female patients, independent influencing factors for MACCE included CRAO (HR 2.196, 95% CI 1.144–4.217, P = 0.018), NLR (HR 1.542, 95% CI 1.196–1.987, P = 0.001), LDL-C (HR 1.759, 95% CI 1.219–2.539, P = 0.003) and hs-CRP (HR 1.019, 95% CI 1.011–1.027, P < 0.001) according to multivariate Cox analysis (Fig. 7).

### Sex differences in the characteristics and prognosis of CRAO patients

The characteristics of male and female CRAO patients are presented in Table 2. The results indicated that male patients with CRAO were more likely to be current smokers (P < 0.001) and current drinkers (P = 0.040), and they were more likely to have higher neutrophil counts (P = 0.045), creatinine (P < 0.001), and UA (P = 0.049), whereas female patients tended to have diabetes (P = 0.037) and higher platelet counts (P = 0.011), and they were more prone to the use of aspirin (P = 0.002) and statins (P = 0.001).There were no sex differences in the incidences of MACCE (all P > 0.05). Kaplan–Meier analysis indicated that there was no significant difference in the event-free survival rate between male and female CRAO patients (χ^2^ = 0.539, P = 0.463).

**Table 2:** Characteristics of male and female patients with central retinal artery occlusion.

	Male (n = 67)	Female (n = 57)	t/Z/χ^2^	P
Age (years)	61.25 ± 11.72	62.06 ± 11.68	0.379	0.705
Hypertension (%)	25 (37.3)	30 (52.6)	2.928	0.087
Duration of hypertension (years)	8.00 (5.00, 10.00)	6.50 (3.00, 11.25)	0.802	0.423
Diabetes (%)	7 (10.4)	14 (24.6)	4.361	0.037*
Duration of diabetes (years)	10.00 (5.00, 15.00)	5.00 (2.00, 10.00)	1.353	0.176
Current smoker (%)	21 (31.8)	3 (5.3)	13.733	< 0.001*
Duration of smoking (years)	30.00 (11.50, 30.00)	30.00 (14.00)	0.629	0.529
Current smoking cigarettes (per day)	16.00 (8.00, 20.00)	10.00 (5.00)	0.544	0.586
Current drinker (%)	9 (13.4)	1 (1.8)	4.200	0.040*
Fatty liver (%)	3 (4.5)	2 (3.5)	0.000	1.000
WBC (× 10^9^/L)	6.37 ± 2.07	5.77 ± 1.49	1.839	0.068
Neutrophil (× 10^9^/L)	4.17 ± 1.63	3.63 ± 1.27	2.022	0.045*
Lymphocyte (× 10^9^/L)	1.7 ± 0.67	1.67 ± 0.54	0.269	0.789
NLR	2.27 (1.69, 3.03)	2.10 (1.50, 2.83)	1.767	0.077
PLT (× 10^9^/L)	208.66 ± 55.17	221.99 ± 61.38	2.547	0.011*
MPV (fL)	11.29 ± 1.99	11.40 ± 2.12	0.590	0.555
PDW (%)	12.7 ± 2.85	12.9 ± 2.13	0.444	0.658
PLR	129.22 ± 47.05	129.49 ± 44.11	0.065	0.948
Creatinine (μmol/L)	75.30 ± 14.76	57.57 ± 14.21	13.595	< 0.001*
eGFR (mL/min)	92.19 ± 14.27	93.66 ± 14.59	1.132	0.258
UA (μmol /L)	411.03 ± 105.06	374.23 ± 100.52	1.983	0.049*
Glucose (mmol/L)	5.39 ± 1.52	5.05 ± 0.86	1.488	0.139
TG (mmol/L)	1.54 (1.18, 2.36)	1.64 (1.26, 2.45)	0.684	0.494
TC (mmol/L)	4.68 ± 1	4.87 ± 1.11	0.990	0.324
HDL-C (mmol/L)	1.13 ± 0.32	1.14 ± 0.3	0.272	0.786
LDL-C (mmol/L)	2.76 ± 0.78	2.69 ± 0.97	0.452	0.652
Apo A1 (g/L)	1.33 ± 0.22	1.38 ± 0.24	1.084	0.280
Apo B (g/L)	0.96 ± 0.2	0.93 ± 0.19	0.980	0.329
Lp (a) (g/L)	167.30 (83.40, 482.00)	120.00 (72.60, 256.70)	1.810	0.070
hs-CRP (mg/L)	1.09 (0.31, 4.21)	1.30 (0.40, 3.68)	0.083	0.934
Fibrinogen (g/L)	2.9 ± 0.71	3.05 ± 0.87	1.093	0.277
TBil (μmol/L)	15.43 ± 5.77	14.83 ± 6.95	0.519	0.605
DBil (μmol/L)	4.23 ± 1.84	4.33 ± 2.48	0.250	0.803
Medications (%)				
Aspirin	31 (46.3)	42 (73.7)	9.560	0.002*
Statins	40 (59.7)	49 (86.0)	10.486	0.001*
β-Blocker	16 (23.9)	12 (21.1)	0.141	0.707
ACEI/ARB	7 (10.4)	8 (14.0)	0.373	0.542
CCB	14 (20.9)	12 (21.1)	0.001	0.983

*WBC* white blood cell count, *NLR* neutrophil‑to‑lymphocyte ratio, *PLT* platelet count, *MPV* mean platelet volume, *PDW* platelet distribution width, *PLR* platelet‑to‑lymphocyte ratio, *eGFR* estimated glomerular filtration rate, *UA* uric acid, *TG* triglycerides, *TC* total cholesterol, *HDL-C* high-density lipoprotein cholesterol, *LDL-C* low-density lipoprotein cholesterol, *Apo A1* Apolipoprotein A1, *Apo B* Apolipoprotein B, *Lp (a)* lipoprotein a, hs-CRP hypersensitive C-reactive protein, *TBil* total bilirubin, *DBil* direct bilirubin, *ACEI* angiotensin converting enzyme inhibitor, *ARB* angiotensin receptor blockers, *CCB* calcium ion channel blockers. *Indicates P < 0.05.

## Discussion

This retrospective cohort study showed that CRAO patients had higher NLR and hs-CRP levels than control group and CRAO was an independent risk factor for MACCE. The CRAO-sex interaction increased the risk of MACCE. Sex-specific analyses indicated that CRAO remained a potential independent predictor of the occurrence of MACCE in both sexes. Age, diabetes and current smoking were the male-specific risk factors for MACCE, while LDL-C was the female-specific risk factor. There was no significant difference in the incidence of MACCE or MACCE-free survival rates between male and female patients with CRAO.

CRAO and cardiovascular and cerebrovascular diseases share atherothrombosis as a pathological characteristic and hypertension, diabetes, and hyperlipidemia as systematic risk factors; therefore, CRAO may serve as a predictor of poor prognosis and the incidence of MACCE. A retrospective study revealed that the degree of atherosclerosis in the retinal vessels was closely related to MACCE occurrence in 436 ACS patients^19^. Moreover, previous studies showed that RAO was closely related to a significantly greater risk of ACS, especially in patients in the high-risk subgroups^5^. However, to the best of our knowledge, the underlying association between CRAO and MACCE has not been quantified and compared after adjusting for important demographic characteristics, such as alcohol consumption, smoking history, medications and parameters of blood cell counts, and biochemical parameters. In current study, we found that CRAO remained an important poor prognostic factor for MACCE after correcting for potential confounding factors, including inflammatory indicators, and lipid profiles. In 2002, Peter Lanzer and Eric J. Topol first introduced the concept of "panvascular disease", which considers the vascular system as a whole and indicates that blood vessels share structural and functional similarities, similar risk factors and prevention and control measures. Therefore, our study has given further support for the panvascular concept of interdisciplinary integration and the research pattern of multidisciplinary coordination.

Currently, knowledge of the pathological mechanism of atherosclerosis is mainly concentrated on the "inflammatory hypothesis"^20^. Therefore, mounting literature acknowledges the importance of assessing the cross-links between biomarkers of inflammation and MACCE risk^21,22,23^. Moreover, NLR and hs-CRP, which are able to reflect inflammation, may be involved in the development of atherosclerosis and thus are more likely to induce cardiovascular and cerebrovascular disease^21,22,23^. Past research has also revealed that there was a correlation between NLR and increased mortality in AMI patients^24,25^. Moreover, the NLR may correlate with the severity of atherosclerosis^21^. In addition, previous studies demonstrated that hs-CRP was closely associated not only with the degree of atherosclerosis but also with a high risk of developing MACCE^26,27^. Our studies indicated that patients with CRAO were more likely to have higher NLR and hs-CRP levels than control group. Furthermore, we uncovered the CRAO patients were in a state of hyperinflammatory state as the disease progressed and, subsequently, a worse clinical outcomes. Thus, inflammation plays a crucial role in the potential mechanism of CRAO and contributes to a high risk of developing MACCE.

Sex-specific research on cardiovascular risk factors has revealed that although females and males share common risk factors for CAD, there are still risk factors that are associated with cardiovascular disease in sex-specific populations. Also, we found that the significant CRAO-sex interaction in all of the evaluated patients provides a significant clue of explanation for the heightened MACCE risk in patients with CRAO. Further subgroup analyses were performed for sex, and we found that CRAO remained a potential independent predictor of poor outcomes in both sexes. Investigating the relationship between gender and CRAO may help to clarify whether the condition has effects on cardiovascular health that are particular to either gender. This could then aid in the creation of gender-specific preventative and treatment plans, deepen our understanding of cardiovascular illnesses, and set the groundwork for more extensive research. Moreover, we found that LDL-C was an independent risk factor for MACCE in female patients. On the one hand, possibly more importantly, this finding may be due to the rapid decline in estrogen in postmenopausal women because estrogen is an important vasoprotective factor that inhibits the endocytosis of LDL-C by vascular endothelial cells and reduces lipid deposition in the vascular wall^28^. On the other hand, the incidence of dyslipidemia in postmenopausal women increases significantly, and LDL-C levels in women over 50 years old are significantly higher than those in men of the same age in China^29^. Recent findings have also indicated that the age-dependent increase in LDL-C is greater in women than men^30^ and the above mechanisms may collectively contribute to LDL-C as a female-specific risk factor for MACCE. Diabetes elevates cardiovascular risk, which has been confirmed by a series of studies^31,32^. Furthermore, there are many potential and documented effects of sex on MACCE, including heart failure, stroke, and myocardial infarction^33^. According to our study, diabetes was a male-specific independent risk factor for MACCE, which is contrary to the latest relevant findings^34^ and the specific mechanisms deserved further study. In addition, our study supports that current smoking is an independent risk factor for MACCE in male patients. Thus, risk factors for MACCE differed between males and females, suggesting that effective prevention of the occurrence of MACCE in high-risk patients requires that attention be given to individualized risk factors corresponding to different sexes. There was no significant difference in the incidence of MACCE or MACCE-free survival rates between male and female patients with CRAO, indicating the similar prognosis of male and female CRAO patients. These findings suggest that we should pay attention to the individualized prevention of cardiovascular risk factors and the management of cardiovascular and cerebrovascular diseases according to sex-specific independent risk factors.

As a retrospective and observational study, the results unavoidably may be disturbed by recall bias. Our findings should be validated in studies with large samples and prospective follow-up. In addition, although a large number of possible confounders have been adjusted for, other risk factors associated with CRAO and MACCE such as the coagulative pattern, the atherosclerosis of the supra-aortic trunks, and the cardiac situation were not been analyzed and even unknown confounders may still exist. Thirdly, sex-related differences in risk factors for MACCE were found in our study. However, different medication use that was found to be gender influenced were not included, such as oral contraceptives and oral menopausal hormone. It has been reported that oral contraceptives increase stroke risk in young women (odds ratio 2.47)^35^ and clinical trials show oral menopausal hormone therapy raises total and ischemic stroke rates^36^. While other latest evidence indicates using low-dose transdermal estrogen effectively treats menopausal symptoms without elevating stroke risk^37^. Although the association between female-specific medication and stroke is controversial, it is interesting to investigate the associations and offer additional insights into sex differences in risk factors for MACCE. Further research on pharmacological influences on MACCE is warranted, especially in female patients with vascular risk factors.

## Conclusions

In conclusions, our study emphasizes the need to focus on CRAO as a potential risk factor for the development of MACCE. The inflammation plays a crucial role in the potential mechanism of CRAO and contributes to a high risk of developing MACCE. Regardless of the presence of other cardiovascular risk factors, a thorough cardiovascular and cerebrovascular evaluation is warranted in CRAO patients. The sex-related differences in risk factors for MACCE suggested that effective prevention of the occurrence of MACCE in high-risk patients requires that attention be given to individualized risk factors corresponding to different sexes.

## Data Availability

The datasets used and/or analysed during the current study available from the corresponding author on reasonable request.
